# Bridging Small Molecules to Modified Bacterial Microparticles Using a Disulphide Linkage: MIS416 as a Cargo Delivery System

**DOI:** 10.1371/journal.pone.0145403

**Published:** 2015-12-22

**Authors:** Francesco Mainini, David S. Larsen, Gill A. Webster, Sarah L. Young, Michael R. Eccles

**Affiliations:** 1 Department of Pathology, University of Otago, Dunedin, New Zealand; 2 Department of Chemistry, University of Otago, Dunedin, New Zealand; 3 Innate Immunotherapeutics Ltd, 4B Walls Rd, Penrose, Auckland, New Zealand; 4 Maurice Wilkins Centre for Molecular Biodiscovery, 3A Symonds Street, Auckland, New Zealand; University of Helsinki, FINLAND

## Abstract

MIS416 is an intact minimal cell wall skeleton derived from *Proprionibacterium acnes* that is phagocytosed by antigen presenting cells, including dendritic cells (DCs). This property allows MIS416 to be exploited as a vehicle for the delivery of peptide antigens or other molecules (for example, nucleic acids) to DCs. We previously showed that covalent (non-cleavable) conjugation of OVA, a model antigen derived from ovalbumin, to MIS416 enhanced immune responses in DCs *in vivo*, compared to unconjugated MIS416 and OVA. Intracellular trafficking promotes the lysosomal degradation of MIS416, leading to the destruction of MIS416 plus the associated cargos conjugated to MIS416. However, lysosomal degradation of cargo may not be desired for some MIS416 conjugates. Here we have investigated whether a cleavable linkage could facilitate release of the cargo in the cytoplasm of DCs to avoid lysosomal degradation. DCs were treated *in vitro* with disulfide-containing conjugates, and as hypothesised faster release of SIINFEKL peptide in the cytoplasm of DCs was observed with the inclusion of a disulfide bond between MIS416 and cargo. The inclusion of a cleavable disulfide bond in the conjugates did not significantly alter the amount of SIINFEKL antigens presented on MHC I molecules on DCs as compared with conjugates without a disulfide bond. However, the conjugates containing disulfide-linkages performed either slightly better (p<0.05) than, or the same as conjugates without a disulfide bond with respect to *in vitro* OT-1 T-cell proliferation induced by the presentation of SIINFEKL antigens on DCs, or DC activation studies, respectively. However, disulfide-containing conjugates were less effective than conjugates without a disulfide bond in *in vivo* cytotoxicity assays. In conclusion, inclusion of a disulfide bond in MIS416-peptide conjugates was associated with efficient release of peptides in the cytoplasm of DCs, an important consideration for MIS416-mediated delivery of degradation-sensitive cargoes. However, treatment of DCs with disulfide-containing conjugates did not significantly alter the presentation of peptide antigens on MHC class I molecules to T-cells, or greatly enhance antigen-associated T-cell proliferation *in vitro*.

## Introduction

MIS416 is a novel vaccine adjuvant-cargo co-delivery system, comprising a micro-particulate formulation of propionibacterium acnes cell wall skeletons consisting of immunostimulatory muramyl dipeptide repeats and nucleic acids [1]. These microparticles rapidly accumulate in DCs and macrophages, which have the capacity to serve as antigen presenting cells (APCs). MIS416 contains nucleotide-binding oligomerization domain containing 2 (NOD-2) and toll-like receptor-9 (TLR-9) ligands, both of which have well-described adjuvant activity [2,3]. Activation of these receptors results in the up regulation of co-stimulatory molecules such as MHC I and II, CD86 and CD80 on APCs [2]. These are essential for the initiation of an effective adaptive immune response in the host. Given its inherent adjuvant properties, MIS416 microparticles could provide an ideal vehicle for co-delivery of cargo such as peptide antigens, as well as other types of immune modulatory nucleic acids and small drug-like molecules to achieve a tailored, single platform adjuvant-cargo co-delivery system targeted to APCs. Webster and colleagues have shown the feasibility of such an approach using the model antigen, OVA, derived from ovalbumin as the target immunogen coupled to MIS416, to enhance adaptive antigen specific immunity [1]. Covalent attachment of antigen was achieved by exploiting amine groups in MIS416 by the formation of activated esters, using N-γ-maleimidobutyryloxysuccinimide ester (sulfo-GMBS) as a linking group between MIS416 amines and OVA associated sulphide groups. Mice immunized with the conjugate showed an increased vaccine response compared to those receiving the same amount of antigen admixed with MIS416 as measured by expansion of OVA-specific CD8+ T cells, and the vaccine response was associated with delayed onset of tumor growth using B16 melanoma cells in a xenograft mouse model, confirming induction of effective anti-tumor immunity [1]. These findings are consistent with the idea that the development of more potent vaccines can be achieved by synchronizing adjuvant and antigen delivery to DCs by methods that link individual vaccine components.

The preceding studies suggested that MIS416 could serve as a delivery platform for a wide range of biomolecular cargos, including degradation-sensitive cargos, such as nucleic acids. However, the above-cited example was dependent on the lysosomal processing of the conjugate to release antigen. To investigate alternatives that might be able to avoid lysosomal processing of the delivered cargo, other linkages were examined. A commonly exploited biological mechanism for drug release is to make use of the intracellular reducing environment of the cytoplasm of cells. The 1000-fold difference in intracellular versus extracellular glutathione concentration (10 mM compared 10 μM) generates a reducing environment in the cytoplasm of the cell that readily cleaves disulfide bonds [4]. Therefore the inclusion of a disulfide bond in the linker between MIS416 and the cargo would result in the cleavage of the disulfide bond by intracellular glutathione [5], releasing the cargo and therefore potentially avoiding the lysosomal degradation pathway during the delivery of the cargo.

**Fig 1:**
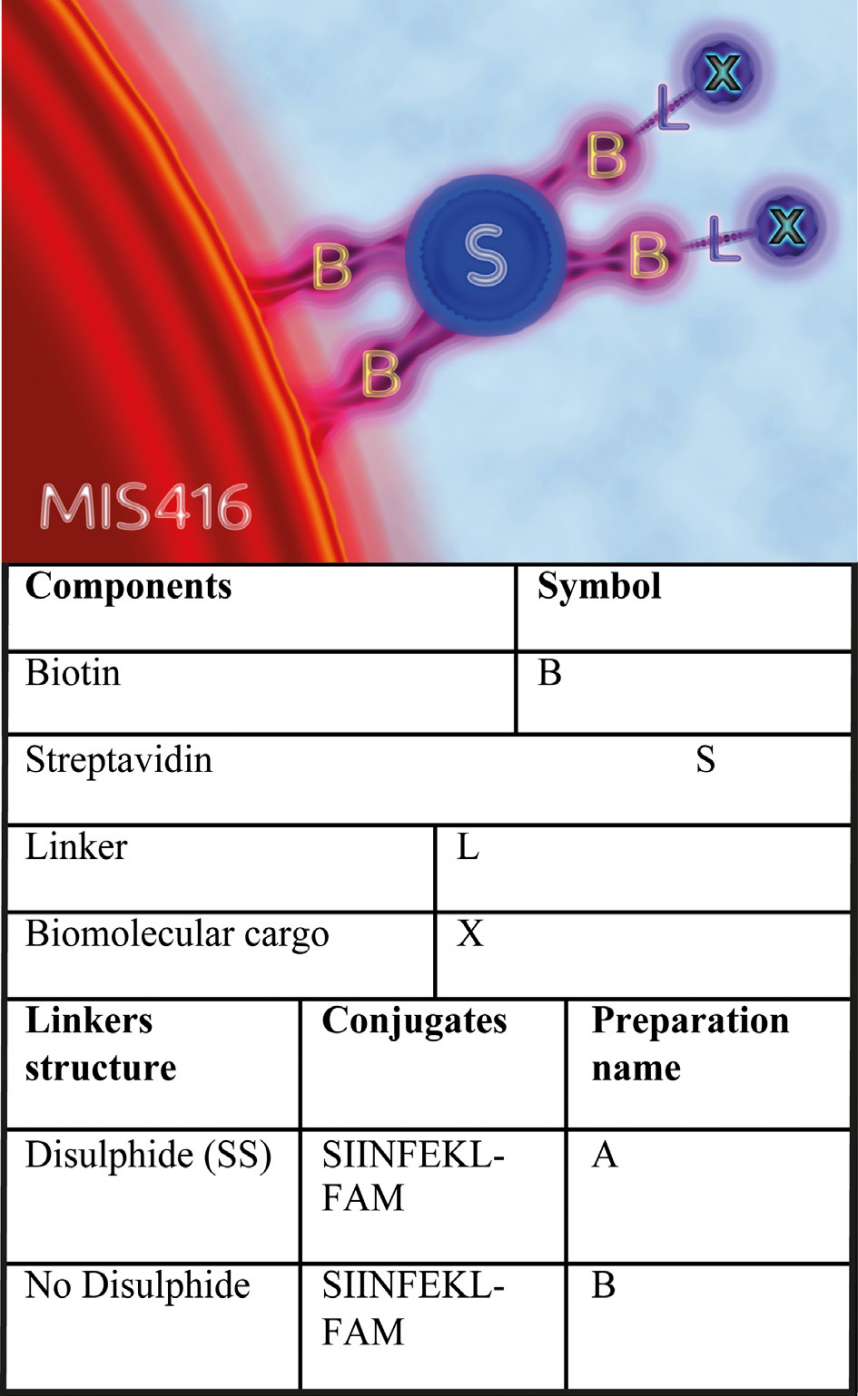
Coupling methodology. The conjugation strategy used to link MIS416 to biotinylated peptides and fluorophores. MIS416 was modified with the addition of biotin (B). Biomolecular cargos (X) were previously biotinylated and then conjugated to MIS416-biotin using streptavidin (S) as a bridge. The addition of a disulfide bond in the linking group (L) facilitates the release of the attached cargos in the cytoplasm of the target cells.

Here we have investigated the hypothesis that inclusion of a cleavable disulfide linkage between MIS416 and SIINFEKL (a small antigenic peptide [serine-isoleucine-isoleucine-asparagine-phenyalanine-glutamine-lysine-leucine] derived from the OVA antigen) would enhance release of the attached cargo in the cytoplasm, and thereby avoid lysosomal degradation of the cargo (which may be useful for some cargos, such as nucleic acids), and modify the level of presentation of SIINFEKL antigen on the surface of DCs following treatment of DCs with MIS416-SIINFEKL conjugates. We have compared two different MIS416 conjugates (see conjugate preparations **A** and **B** in Fig 1) for delivery of the SIINFEKL OVA peptide antigen; one conjugate incorporated a cleavable disulfide bond between MIS416 and the peptide, and the other conjugate contained no disulfide linkage. We determined the percentage of DCs presenting SIINFEKL antigen in MHC class I molecules on the surface of DCs, and incorporated fluorescent labels in the MIS416 conjugates to assess the rate of delivery of SIINFEKL peptide in the cytoplasm of DCs.

## Results

### Coupling of biotinylated molecular cargos to MIS416

**Fig 2:**
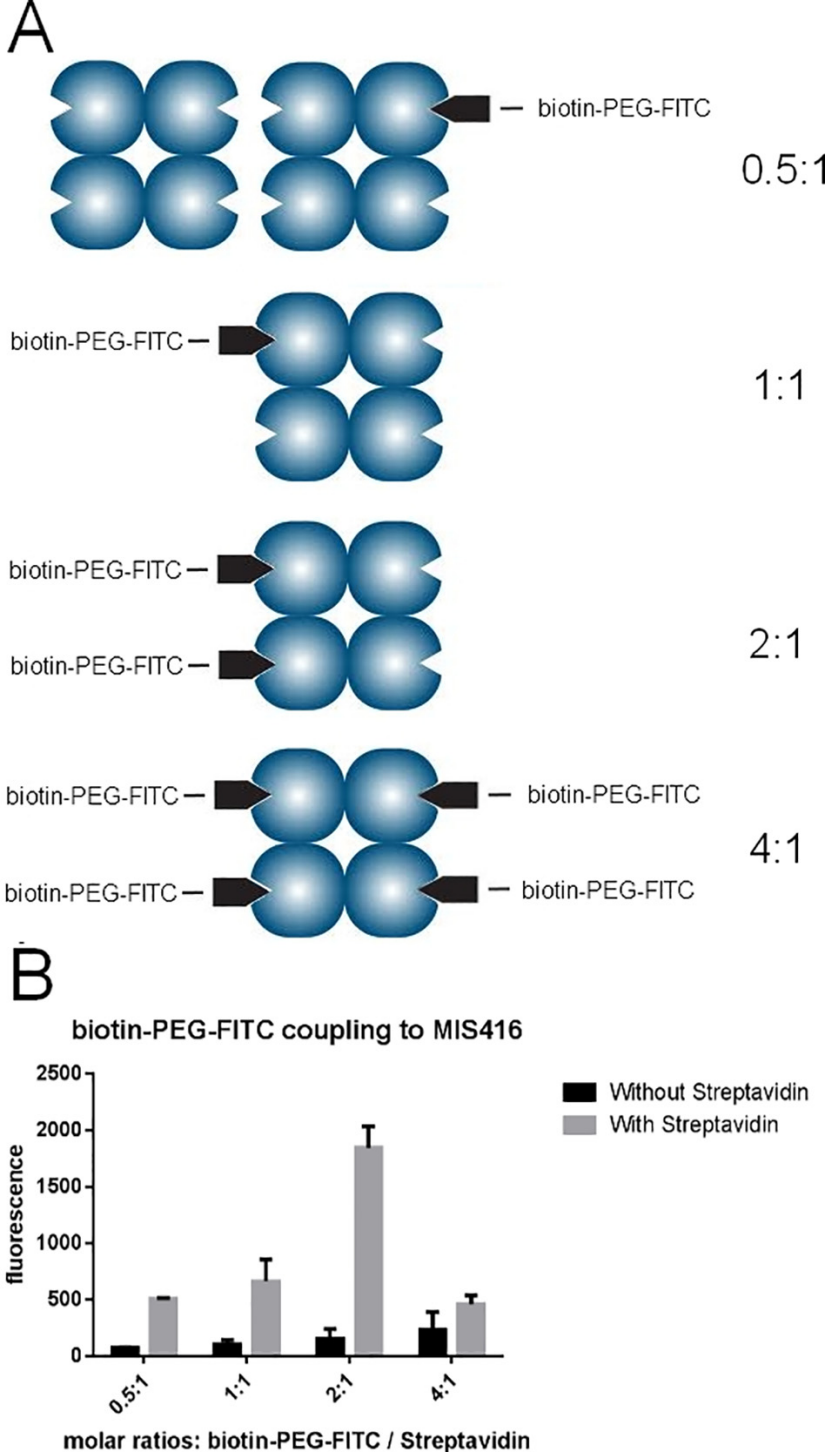
Coupling of biotin-PEG-FITC to MIS416 using a SAV bridge. A) Representation of biotin-PEG-FITC:SAV molar ratios used for the conjugation assay in B. B) Fluorescence output (excitation 488nm, emission 520nm) of different MIS416-biotin-SAV-biotin-PEG-FITC conjugates prepared, using molar ratios of 0.5:1 to 4:1 to occupy the biotin-binding sites on SAV prior to then reacting the SAV-biotin-PEG-FITC molecular complex with MIS416-biotin. Negative controls (black bars) represent the non-specific binding of biotin-PEG-FITC to MIS416-biotin. Error bars represent the standard error of the mean (SEM). This experiment was repeated three times.

The approach we have developed is shown in Figure A in S1 File and relies on streptavidin (SAV) acting as a bridge between the biotinylated bacterial microparticle and a biotinylated small biomolecule. A series of experiments varying the molar ratio of a biotinylated pegylated fluorescein isothiocyanate derivative (biotin-PEG-FITC) to SAV (Table 1, Materials and Methods, Fig 2A) was undertaken. The resulting complexes were mixed in varying molar ratios with MIS416-biotin. As negative controls, and also to measure the background fluorescence, the same amounts of biotin-PEG-FITC were mixed with MIS416-biotin without the addition of SAV. The results showed that when two biotin-binding sites on SAV were occupied with biotin-PEG-FITC (Fig 2B, column 2:1), this SAV-biotin-PEG-FITC complex could then additionally react with MIS416-biotin, and subsequently be isolated as a pellet by centrifugation. The re-suspended pellet was demonstrated to have a relatively high fluorescence. However, when all four binding sites on SAV were saturated with biotin-PEG-FITC (Fig 2B, column 4:1), the SAV-biotin-PEG-FITC complex was not able to react with the MIS416-biotin, and the fluorescence of the resulting pellet was markedly lower than either the 1:1 or 2:1 ratios (Fig 2). These experiments demonstrated that relatively efficient coupling of fluorescent cargos onto MIS416 was achieved using this conjugation strategy.

**Table 1:** Molar ratios used to prepare various MIS416/biotin-PEG-FITC conjugates in Fig 2.

Molar ratio Biotin/SAV	Volume of a 1:10 diluition of biotin-PEG-FITC	Amount of biotin-PEG-FITC used
0.5	0.8 μL	4 μg (1.17 nmoles)
1	1.6 μL	8 μg (2.34 nmoles)
2	3.2 μL	16 μg (4.68 nmoles)
4	6.4 μL	32 μg (9.36 nmoles)

### Cleavage of the disulfide bond facilitated release of the conjugated fluorescent peptide from MIS416 *in vitro*

**Fig 3:**
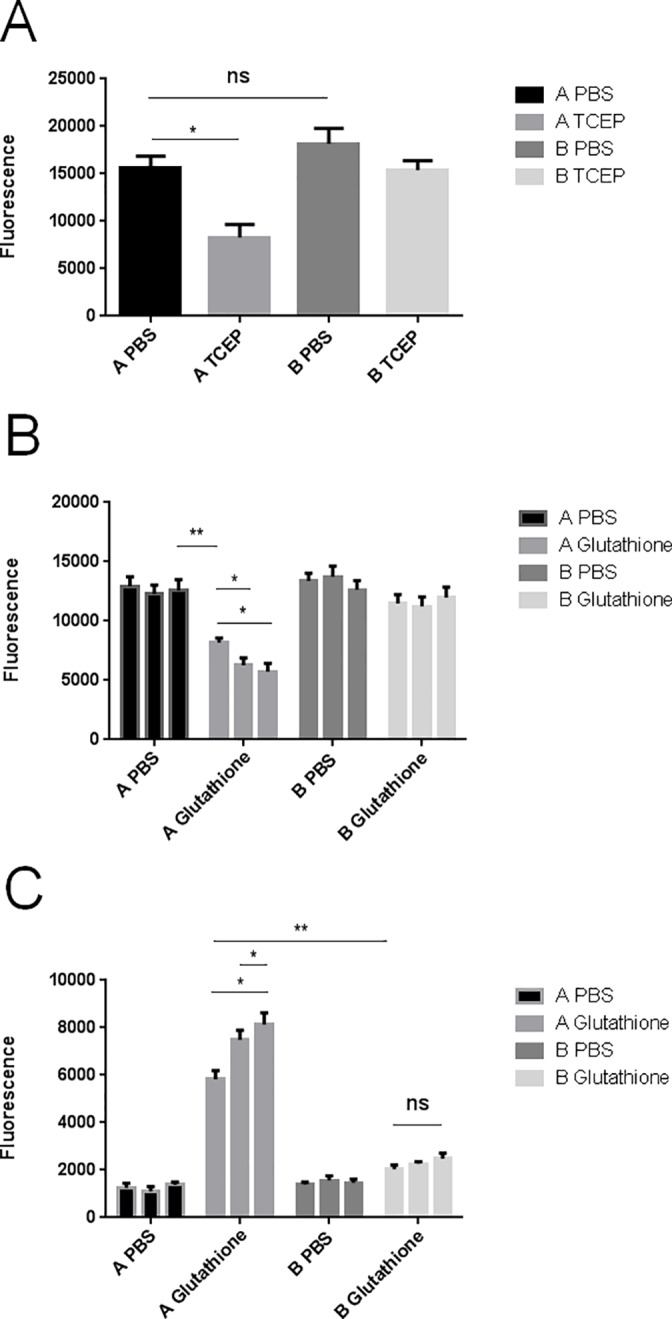
Cleavage of the disulfide bond with TCEP or glutathione *in vitro*. A) Release of fluorescent peptide into solution following cleavage of the disulfide bond in conjugates using TCEP *in vitro*. Conjugates A and B (30 μg) were treated with PBS (100 μL) or TCEP (100 μL, 50mM pH 7) for 30 min at RT. After washes, pellets were re-suspended in PBS (100 μl) and fluorescence was measured (excitation 488, emission 520). Error bars represent SEM. B and C) Release of the fluorescent peptides into solution following cleavage of the disulfide bond in the conjugates using glutathione *in vitro*. Conjugates A and B (30 μg) were treated with PBS (100 μL) or glutathione (100 μL, 10 mM, pH 7.2) for 30, 60 or 120 min at RT as represented by the 3 bars left to right. After centrifugation, pellets (B) and supernatants (C) were collected and the fluorescence measured (excitation 488, emission 520). Error bars represent SEM. All experiments were repeated three times.

The incorporation of a disulfide linkage between MIS416 and the peptide cargo was used to investigate whether rapid release of the attached peptide would occur in the cytoplasm of DCs [4]. To demonstrate that the disulfide bond was potentially cleavable we monitored release of fluorescently labelled peptide from the MIS416 conjugates upon cleavage of the disulfide bond in reducing environments *in vitro*. The two MIS416 conjugates were prepared as in Figure A in S1 File; one that included a disulfide bond between the MIS416 microparticle and attached fluorescent peptide (conjugate **A**, see Fig 1), and a conjugate without the disulfide bond (conjugate **B**, Fig 1). Cleavage of the disulfide bond was carried out by incubating the conjugates for 30 min in 50 mM tris(2-carboxyethyl)phosphine (TCEP), pH 7 at room temperature (RT) (Fig 3A). The results showed that TCEP treatment of conjugate **A** (containing a disulfide bond in the linker) was associated with release of the fluorescent peptide into solution, as compared to little or no release from conjugate **B**, or when the conjugates were treated with phosphate buffered saline (PBS). In addition, treatment of the conjugates with glutathione as a reducing agent for 30, 60 or 120 minutes, to simulate the reducing environment in the cytoplasm of cells, gave an almost identical outcome as treatment with TCEP (Fig 3B and 3C)

### The disulfide bond was cleaved in the cytoplasm of DCs facilitating release of the conjugated fluorescent peptide from MIS416

**Fig 4:**
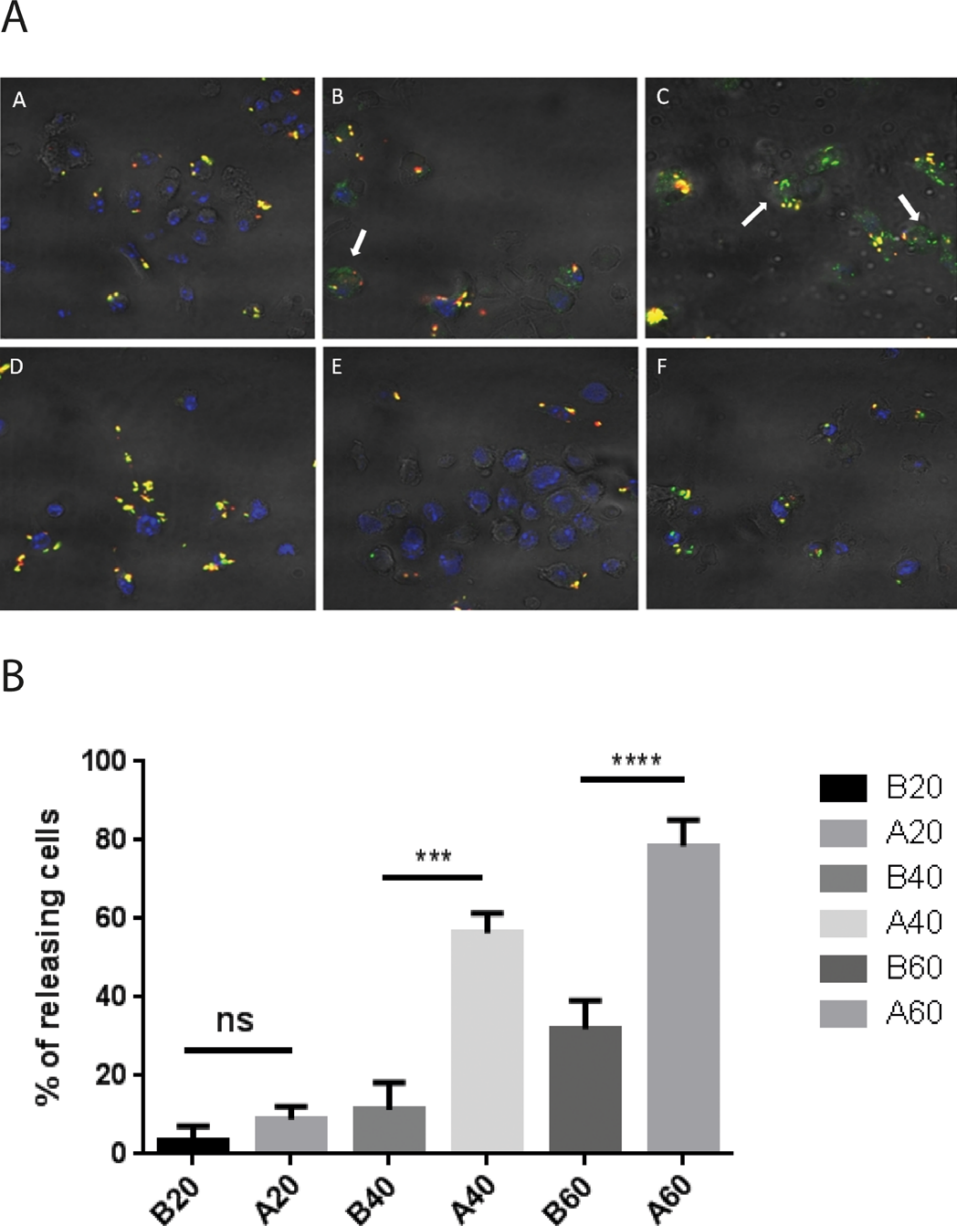
Cleavage of the disulfide bond allowed the release of conjugated FAM-SIINFEKL peptide from MIS416 in the cytoplasm of DCs. A) Confocal microscopy was used to analyse BMDCs treated with MIS416 conjugate A^1^ (MIS416-biotin-SAV-biotin-SS-SIINFEKL-FAM) and B^1^ (MIS416-biotin-SAV-biotin-ttds-SIINFEK-FAM). The top three panels (A, B, C) show fluorescent images of BMDC treated with conjugate A^1^ while the lower panels (D, E, F) are of BMDC treated with conjugate B^1^. Panels A and D represent BMDCs photographed immediately following washing with PBS to remove excess MIS416 conjugates from the medium after 20 min of uptake. B and E represent BMDCs photographed 20 min after washing, while C and F represent BMDCs photographed 40 min after washing. Yellow staining represents co-localisation in BMDCs of SAV-APC-labelled MIS416 with SIINFEKL-FAM. Red staining represents SAV-APC-labelled MIS416 in BMDCs. Green staining represents SIINFEKL-FAM (white arrows) released from MIS416 in BMDCs, and blue staining represents the nuclei of the BMDCs stained with DAPI. B) Graph representing the percentage of releasing cells obtained from confocal microscopy. Cells in the images were scored if they contained at least 1 internalized microparticle (see Materials and Methods). The total number of cells counted for each condition was as follows: A = 24, B = 34, C = 66, D = 23, E = 78, F = 56. Error bars represent SEM.

Next, we carried out experiments to visualize release of SIINFEKL-FAM from conjugates **A** or **B** in the cytoplasm of mouse bone marrow dendritic cells (BMDC). We hypothesized that for conjugate **A**, SIINFEKL-FAM would be released in the cytoplasm, while conjugate **B** would undergo slower release caused only by lysosomal processing. To visualize the MIS416 component once the SIINFEKL-FAM had been released, MIS416 was labelled with streptavidin-allophycocyanin (SAV-APC), making use of the unbound biotin molecules available on the surface of the MIS416 in conjugates **A** and **B**. Following this labelling the new conjugates were referred to as conjugates **A** ^**1**^ and **B** ^**1**^, respectively (see Figure B in S1 File for a schematic of conjugate **A** ^**1**^, and Table B in S1 File, listing all the conjugates used in this study). Conjugates **A** ^**1**^ and **B** ^**1**^ (10μg) were added to BMDC for 20 min to allow uptake and internalization (T0, Fig 4). BMDCs were then washed with PBS to remove the non-internalized MIS416 conjugates, and the cells incubated a further period in fresh media. The BMDCs were then allowed to process the conjugates for an additional 20 or 40 min, and the cells were then processed for confocal microscopy. By immunofluorescence it was observed that the release of fluorescent SIINFEKL-FAM from conjugate **A** ^**1**^ occurred within an interval of 20 to 40 min after uptake, while conjugate **B** ^**1**^ did not show similar evidence for release in the cytoplasm. These data suggest that the disulfide bond was cleaved in the cytoplasm of DCs within 20–40 minutes of internalization.

### Conjugates containing disulfide linkages behaved similarly to conjugates lacking a disulfide linkage in inducing presentation of SIINFEKL antigen in MHC class I on DCs, and in activating DCs *in vitro*, but performed slightly better in *in vitro* T-cell proliferation assays, yet worse in *in vivo* cytotoxicity assays

We hypothesized that the cytoplasmic release of SIINFEKL would interfere with the lysosomal processing and presentation of SIINFEKL antigen on MHC class I molecules [6]. To test this hypothesis, we used an *in vitro* assay to determine whether BMDCs are able to process disulfide-containing MIS416-SIINFEKL conjugates more efficiently, and subsequently present SIINFEKL antigens in MHC class I molecules on the cell surface more rapidly than conjugates lacking a disulfide bond.

**Fig 5:**
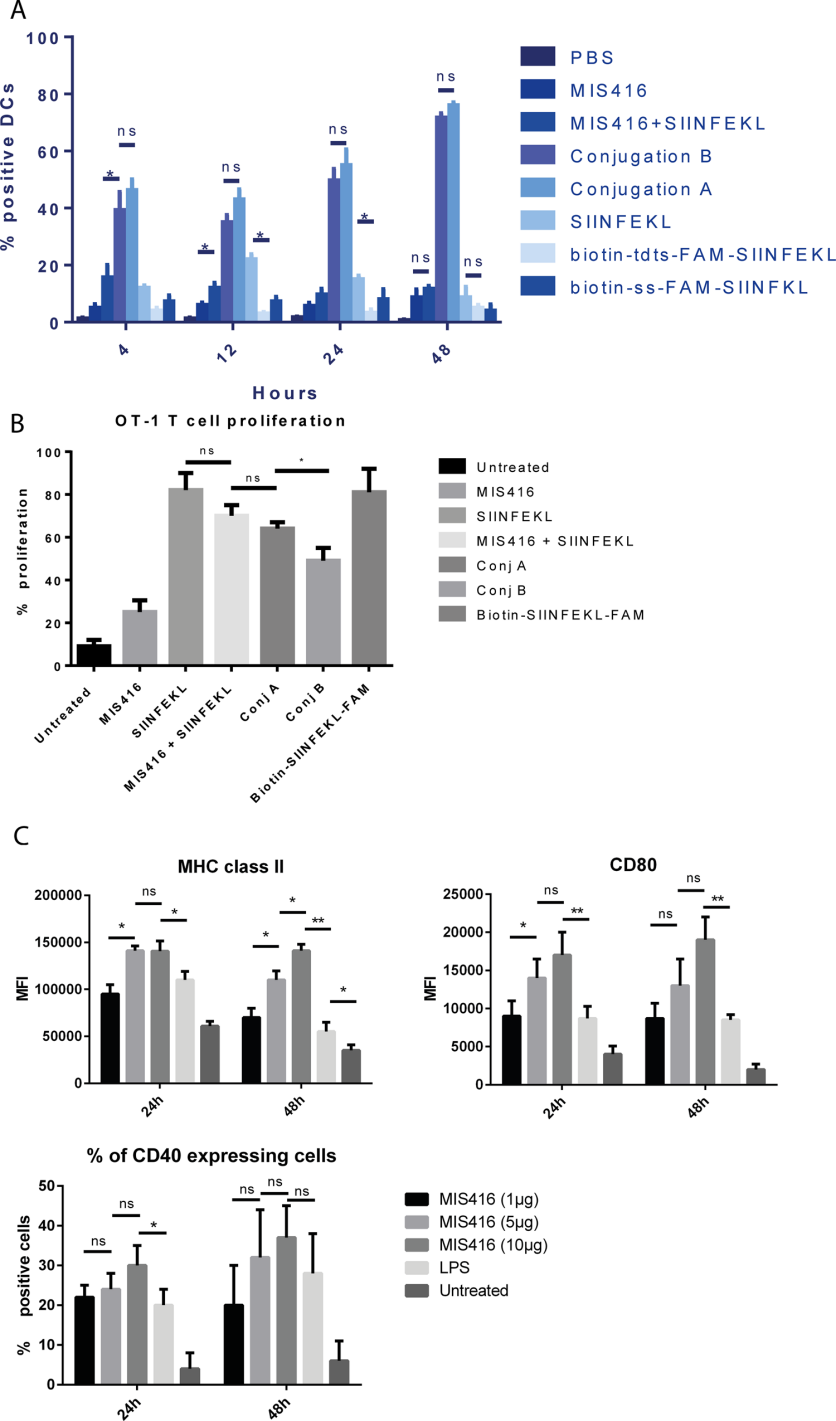
Conjugate A (containing a disulfide bond) behaved similarly to conjugate B (lacking a disulfide bond) with respect to *in vitro* presentation of SIINFEKL on MHC class I molecules on BMDCs, but performed better than conjugate B in *in vitro* T-cell proliferation assays A) BMDCs were treated *in vitro* for 4, 12, 24 and 48h with conjugates of MIS416 and controls, as indicated. BMDCs treated with PBS or MIS416 alone were used to set background fluorescence, and as a negative control. Cells were pre-gated on live cells and positive staining with CD11c. The treatment of BMDCs with SIINFEKL alone, or with a mixture of MIS416 plus SIINFEKL (unconjugated), or biotinylated SIINFEKL (biotin-SS-FAM-SIINFEKL, or biotin-tdts-FAM-SIINFEKL) resulted in approximately 22% of BMDCs presenting SIINFEKL antigen in MHC class I molecules. In contrast, conjugates **A** and **B**, containing a disulfide bond (conjugate **A**), or lacking a disulfide bond (conjugate **B**), were more efficient at processing and presenting SIINFEKL antigen in MHC class I molecules on BMDCs. Conjugate **A** behaved in a similar fashion to conjugate **B**. *, p<0.05. Error bars represent SEM. tdts = 1,13-diamino-4, 7, 10-trioxatridecan-succinic acid. B) DCs (1x10^6^ in 2 mL of media) were treated with LPS (1 μg) and MIS416 (1, 5, 10 μg). Cells were collected and stained in live/dead assays (0.05 μL plus 100 μL of PBS for each sample) and with different antibodies to detect activation markers (CD40, CD80, MHC class II) and DCs (CD11c). Y axis represents the Mean Fluorescence Intensity (MFI). For CD40 the % of positive cells for CD40 was used instead of MFI. C) OT-1 T cell proliferation assays carried out to determine the percentage of proliferating T-cells following exposure to BMDCs. BMDCs were treated with MIS416, SIINFEKL, MIS416 + SIINFEKL, conjugates **A** and **B** and biotin-SIINFEKL-FAM for 24h. Untreated cells were used as negative control. After 24 h, OT-1 T-cells were co-cultured with the treated BMDCs for 72h. The percentage of T-cells proliferating for each sample was calculated using FlowJo (V9) flow cytometry data analysis software. The results represent the combined analysis of 5 separate OT-1 T-cell prolideration assays. Error bars represent SEM. Results that are not significant are marked with ns while significant results are marked with * depending on the P values (* P < 0.05, ** P < 0.005, *** P < 0.0005). This experiment was repeated three times.

We found that pulsing BMDCs with SIINFEKL alone resulted in approximately 13% of DCs presenting the SIINFEKL antigen on the cell surface at 4 h and 20% at 12h, which slowly reduced over the next 36 h (Fig 5A). As expected, compared to BMDCs treated with SIINFEKL alone, BMDCs pulsed with conjugate **B** (MIS416-SIINFEKL without a disulfide bond) exhibited a higher level of SIINFEKL on MHC class I at earlier time points (4 and 12 h, 40% and 37% respectively). However, BMDCs pulsed with conjugate **A** (containing the disulfide bond) were very similar, and were not significantly different to conjugate **B** (4 and 12 h, 52% and 45% respectively) in both the level and the timing of processing of SIINFEKL antigen on MHC class I molecules in DCs. To determine whether MIS416 treatment activates DCs, we assessed the up-regulation of activation markers on BMDCs pulsed *in vitro* with different concentrations of MIS416 or LPS for 24 or 48h. Up-regulation of MHC class II, CD80 and CD40 on DCs was observed following treatment (Fig 5B), the degree of which varied for each activation marker tested, although up-regulation was directly correlated with the concentration of MIS416 used, suggesting that MIS416 was able to activate DCs. The expression of the activation markers was also evaluated following the interaction of BMDCs with conjugates **A** and **B** for 24h to determine whether modification of the MIS416 would have an effect on DC activation, and no significant differences were observed (Figure F in S1 File).

To explore the effect of SIINFEKL antigen presentation on MHC class I molecules in DCs on T-cell activation *in vitro*, we carried out *in vitro* T-cell proliferation assays with conjugates **A**, **B** and controls. The results of the *in vitro* T-cell proliferation assays showed that conjugate **A** was slightly better than Conjugate **B** (p<0.05) at inducing T-cell proliferation (Fig 5C).

**Fig 6:**
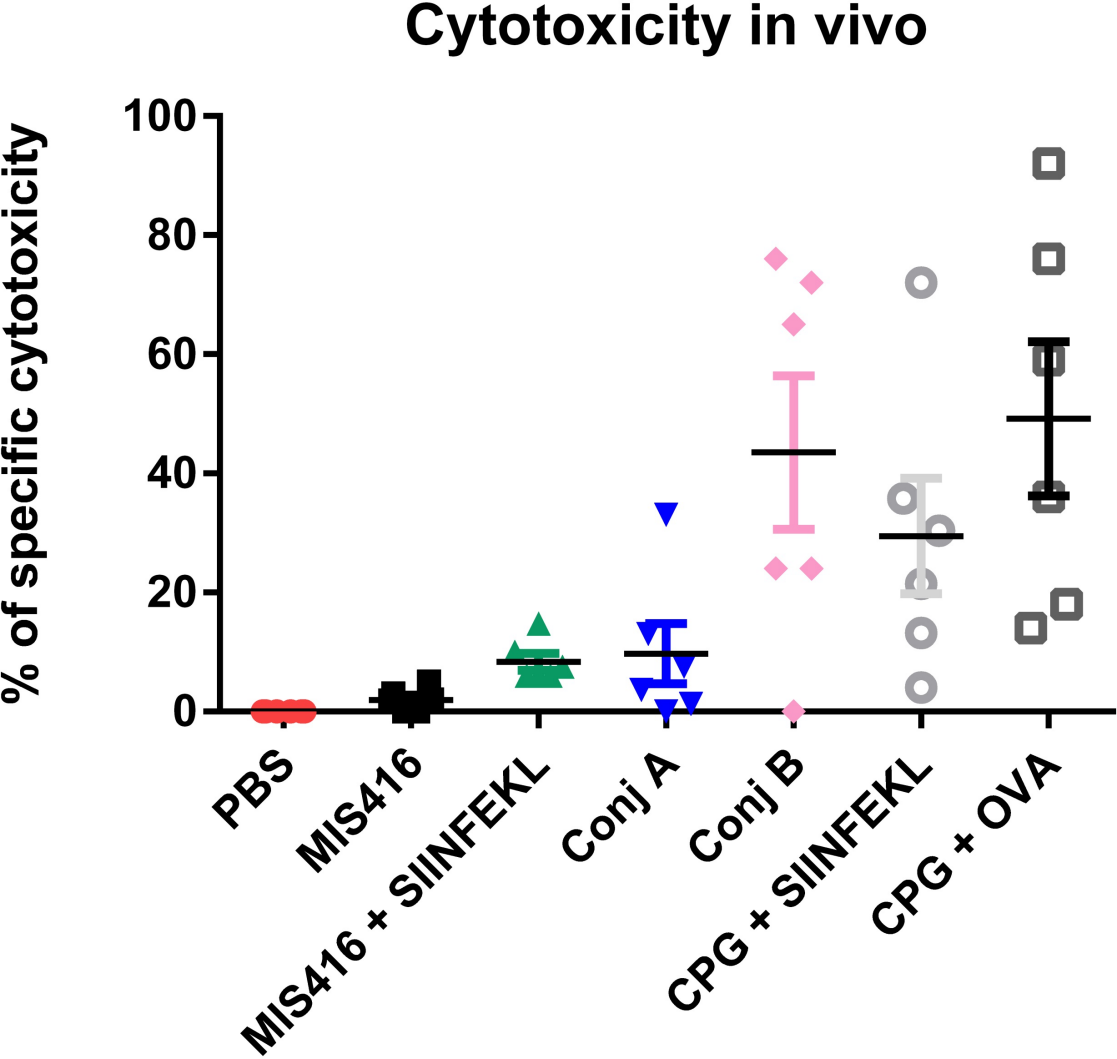
Evaluation of specific cytotoxicity induced by vaccination with MIS416/SIINFEKL conjugates. Six mice were used in each group and they were vaccinated with Conjugation A or B (100 μg), MIS416 (100 μg) plus SIINFEKL (2 μg), MIS416 alone (100 μg), PBS (100 μL), CpG plus SIINFEKL (2 μg) or CpG plus Ovalbumin (10 μL of OVA + 50μg of CpG). After 1 week mice were challenged with two population of splenocytes (1 x 10^7^ cells, pulsed with SIINFEKL or not) and after two days specific cytotoxicity was evaluated. CpG + OVA was used as positive control while PBS and MIS416 were negative controls. Specific cytotoxicity as a percentage was calculated using the ratio between cells stained with VPD450 (pulsed with SIINFEKL) and with CFSE (unpulsed), of the vaccinated groups compared to the control group (PBS).

Finally, to determine whether MIS416/SIINFEKL conjugates have the potential to induce an antigen-specific anti-tumour immune response, an *in vivo* cytotoxicity assay was undertaken. The vaccination efficacies of conjugate **A** (disulfide bond) and conjugate **B** (no disulfide bond) were compared alongside negative (PBS and MIS416) and positive controls (CpG + SIINFEKL or CpG + OVA) (Fig 6). Overall, conjugate **B** was found to be more effective at inducing specific cytotoxicity than conjugate **A**, suggesting that the disulfide bond in conjugate **A** may be unstable *in vivo*, since the efficacy of this conjugate was similar to MIS416 + SIINFEKL (unconjugated). In contrast, vaccination with conjugate **B** was similar to the positive controls (CpG+SIINFEKL or CpG+OVA) (Fig 6).

## Discussion

One of the most exploited bioconjugation strategies to conjugate molecules to microparticles involves the use of bi-functional linkers such as GMBS [7] or SMCC [8]. These linkers require an amine on one of the components, and either an amino or thiol group on the other [9,10], in order to allow the formation of bonds that are not readily cleaved in the cell. MIS416-cargo conjugates containing bonds such as these are processed in the lysosomal pathway, which then leads to lysosome-mediated degradation of both the MIS416 and cargo. The strategy developed in this research sought to use an alternative linkage to allow cleavable bonds for attaching conjugation partners to MIS416. To achieve this, a conjugation strategy involving SAV-biotin was devised, enabling inclusion of a disulfide bond between MIS416 and the cargo. SAV has been used previously in conjugations to couple multiple biomolecules [11] [12]. For example, Chu and colleagues (2006) mixed two different biotinylated compounds to SAV using a 2:2:1 molar ratio, resulting in siRNA-SAV-aptamer conjugates. Our coupling procedure comprised two steps, was rapid and efficient, and was carried out in an aqueous solution, minimizing aggregation and resulting in a relatively homogeneous conjugate. This coupling strategy could potentially be adapted for the conjugation of almost any biotinylated biomolecule to MIS416. The SIINFEKL peptide was chosen as the cargo in this study because there are numerous tools available to measure its fate in DCs [13,14].

MIS416 is an efficient delivery system for DCs, because it is avidly phagocytosed by DCs, and contains the adjuvant ligands, TLR9 and NOD-2 [1]. Therefore, the introduction of a cleavable disulfide bond between SIINFEKL and MIS416, resulting in the release of cargo in the cytoplasm, could potentially be adapted for the delivery of other cargos [15,16], such as for example biotinylated siRNAs, where degradation of the RNA duplex in the lysosomal compartment of target cells would be undesirable.

We observed efficient release of SIINFEKL-FAM from conjugates in the cytoplasm containing a disulfide bond, compared to conjugates lacking the disulfide bond, but only a minor difference was observed in the percentage of DCs presenting SIINFEKL in MHC class I molecules on the cell surface, suggesting that presentation of SIINFEKL antigens on the cell surface of DCs was not significantly affected by release of SIINFEKL in the cytoplasm. Similar results were observed regarding the expression of activation markers on the surface of DCs after the treatment of MIS416 and MIS416/SIINFEKL conjugates. However, conjugate **A** performed slightly better than conjugate **B** in OT-1 T-cell proliferation assays *in vitro*, which could be explained by a minor (though not significant) increase in the generation of MHC/SIINFEKL complexes and in the increased expression of activation markers associated with treatment of DCs with conjugate **A** compared to conjugate **B**. These two minor effects were evident at early time points and could have impacted the exponential growth of OT-1 T cells over the 3 days of the assay resulting in a significant difference in proliferation.

In conclusion, the presence of a disulfide bond in MIS416 conjugates between MIS416 and its associated cargo was associated with the efficient release of the cargo in the cytoplasm of DCs, and the avoidance of lysosomal degradation of the cargos upon delivery to DCs, which is an important consideration when MIS416-mediated delivery of degradation-sensitive cargos, such as nucleic acids, might be undertaken. Following treatment of DCs with MIS416-SIINFEKL conjugates, the presentation of antigens on MHC class I molecules on DCs was not significantly altered, although processing of disulfide-containing MIS416-SIINFEKL conjugates was able to induce T-cell proliferation slightly more efficiently than MIS416-SIINFEKL conjugates lacking the disulfide bond. However, this effect was not confirmed by further *in vivo* experiments where conjugate **B** performed better than conjugate **A** in the generation of a specific immune response against the model peptide antigen SIINFEKL.

## Materials and Methods

### Ethics statement

This study was carried out in strict accordance with the recommendations of the National Animal Ethics Advisory Committee (NAEAC) of New Zealand. The protocol was approved by the Animal Ethics Committee of the University of Otago (Permit Number: ET10/13). Mice used in this study were euthanased by cervical dislocation. All efforts were made to minimize suffering.

### Biotinylation of MIS416

Pellets of MIS416 (10 mg) (Ref. 1) were washed in NaHCO_3_ (sodium bicarbonate) buffer (50 mM, pH = 8.35, 1.5 mL). Sulfo-NHS-biotin (ThermoScientific) (1.2 mg) was dissolved in NaHCO_3_ buffer (50 mM, pH 8.35, 1 mL) and added to the washed pellet. The mixture was agitated overnight. The supernatant was removed after centrifugation (5000 x g, 5 minutes (min)) and the pellet washed three times with PBS buffer (1.5 mL).

The conjugation of biotin to the particle was assessed using SAV-phycoerythrin (SAV-PE), which was purchased from Biolegend as a solution in PBS (0.2 mg/mL). A standard curve of fluorescence versus SAV-PE concentration is shown in Figure C in S1 File. The biotinylated microparticle (1 mg) was suspended in PBS (200 μL). An aliquot of the SAV-PE stock solution (20 μL, 4 μg) was added and the mixture agitated for 3 h. After centrifugation and washing with PBS the pellet was re-suspended in PBS (200 μL). An aliquot (20 μL) was placed in one well of a 96 well plate, and PBS (80 μL) was added. The fluorescence (excitation 500nm, emission 570nm) was measured (Figure D in S1 File).

### Preparation of the MIS416-biotin–SAV–biotin-PEG-FITC conjugate

SAV (Invitrogen, 2.5 mg/mL in PBS) was used to conjugate MIS416-biotin particles and biotin-PEG-FITC (Nanocs, MW 3400g/mol). A stock solution of biotin-PEG-FITC in PBS (50 mg/mL) was prepared from which a working solution in PBS (5 mg/mL) was made. SAV (50 μL, 0.125 mg, 2.37 nmoles) was added to different amounts of biotin-PEG-FITC (See Table 1) in an eppendorf tube in order to occupy 0.5, 1, 2 and 4 of the four biotin-binding sites of SAV. The different mixtures were agitated for 4 h at room temperature.

MIS416-biotin (0.4 mg in 200 μL in sodium bicarbonate buffer, pH 8.3) was added to the four different mixtures and agitated overnight at room temperature. After centrifugation (5000 x g, 5 min) and washing with PBS, the pellets were re-suspended in PBS (200 μL). An aliquot (20 μL) of each sample was then added to PBS (80 μL) and the fluorescence was measured (excitation 488nm, emission 520nm). The experiment was repeated three times with the same batch of MIS416-biotin and the results are summarized in Fig 2. As a control, MIS416-biotin (0.4 mg) in NaHCO_3_ buffer (200 μL, pH 8.3) was added to the biotin-PEG-FITC from the working solution (4, 8, 16 and 32 μg) used in the previous reactions, but without the addition of SAV, to determine the non-specific attachment of biotin-PEG-FITC to MIS416-biotin and set the background fluorescence.

### Preparation of MIS416-biotin-SAV-biotin-SS-SIINFEKL-FAM (A) and MIS416-biotin-SAV-biotin-ttds-SIINFEKL-FAM (B) conjugates

To prepare conjugate **A**, Biotin-SS-SIINFEKL-FAM (JPT Peptide Technologies MW: 1839.15 g/mol) was dissolved in dimethyl sulfoxide (1 μg/μL). Aliquots of this (16 μL, 8.64 nmol) were added to SAV (100 μL, 0.250 mg, 4.74 nmol) in an eppendorf tube to occupy two biotin-binding sites of SAV. The mixture was agitated for 4 h at room temperature. MIS416-biotin (0.2 mg in 100μL in PBS) was added and the mixture agitated overnight at room temperature. After centrifugation (5000 x g, 5 min) the pellet was collected and washed three times with PBS (1.5 mL). The pellet was re-suspended in PBS (200 μL) and stored at 4°C.

To prepare conjugate **B**, Biotin-ttds-SIINFEKL-FAM (JPT Peptide Technologies MW: 2279 g/mol) was dissolved in dimethyl sulfoxide (1 μg/μL), and the same procedure as above was used to prepare MIS416-biotin-SAV-biotin-ttds-SIINFEKL-FAM. In conjugate **B**, the disulfide linker has been substituted with N-(3-{2-[2-(3-amino-propoxy)-ethoxy]-ethoxy}-propyl)-succinamic acid (ttds).

Standard curves of fluorescence versus concentration of biotin-SS-SIINFEKL-FAM) and biotin-ttds-SIINFEKL-FAM were used to evaluate the conjugation efficiencies of the above reactions (Figure E in S1 File). Aliquots (20 μL) of the re-suspended conjugates **A** and **B** were added to PBS (80 μL) and the fluorescence measured (excitation 488nm, emission 520nm). The results were then compared to the calibration curves to estimate the amount of FAM-SIINFEKL that was conjugated to MIS416 (see Table A in S1 File).

### Cleavage of the disulfide bond in MIS416 conjugates *in vitro*

Solutions of conjugates **A** and **B** were prepared as above using a 2:1 molar ratio of biotin-PEG-FITC and SAV. After centrifugation and washing in PBS, pellets were re-suspended in PBS (200 μL). Aliquots (50 μL) were treated with TCEP (tris(2-carboxyethyl)phosphine) (SIGMA, 50mM, pH 7, 100 μL) for 30 min at 37°C, or with glutathione (L-glutathione reduced, Sigma Aldrich, 10mM, pH 7.2, 100 μL) for 30, 60 and 120 min at 37°C. Supernatants were removed after centrifugation (5000 x g, 5 min), and pellets washed three times with PBS buffer (1.5 mL). Pellets were re-suspended in PBS (100 μL) and fluorescence measured (excitation 488 nm, emission 520 nm). Fluorescence of the supernatant (100μL) was also measured following glutathione treatment. An aliquot (50 μL) of the two conjugates was treated with PBS (200 μL), instead of TCEP or glutathione and processed in the same way as a control to evaluate the background loss of fluorescence caused by the three washes. All experiments were repeated three times.

### Bone marrow-derived dendritic cell (BMDC) preparations

Bone marrow was harvested from the femurs and tibias of C57BL/6 mice and red blood cells were lysed with ammonium chloride buffer (4.15 g NH_4_Cl, 0.5 g KHCO_3_, 0.0186 g EDTA, 500 mL milli-Q water, pH 7.4) for 2 min at 37 4°C. Bone marrow cells were then plated at 3x10^6^ cells/well in a 6-well plate with 5 mL of IMDM medium (Invitrogen) supplemented with 5% fetal bovine serum (Moregate), and 5 × 10^−5^ M 2-mercaptoethanol (Gibco,life technologies) and cultured for 6 days in the presence of 20 ng mL^-1^ recombinant granulocyte-macrophage colony-stimulating factor (GMCSF) (Prospec). Every 3 days, half of the cell culture media of the BMDC cultures was replaced with fresh media.

### Visualization of cleavage of the disulfide bond in the cytoplasm of BMDCs by confocal microscopy

Conjugates **A** and **B** were modified with SAV-APC (Biolegend). Solutions of conjugates **A** and **B** (100 μg) in PBS (100 μL) were added to SAV-APC (20 μL 4 μg) to produce conjugates A^1^ and B^1^. The mixtures were agitated for 4 h at room temperature. After centrifugation (5000 x g, 5 min) the pellet was collected and washed with PBS buffer (1.5 mL) three times. The pellet was then resuspended in PBS (100 μL) and stored at 4°C.

2x10^6^ BMDCs at day 6 were plated in a 6 well plate with 4 coverslips (13 mm diameter) in each well with 3 mL of complete media. BMDCs were allowed to adhere to the coverslips overnight. The next day single coverslips with BMDCs were placed in a 24 well plate with complete media (250 μL) and the cells were pulsed with modified conjugates **A** ^**1**^ or **B** ^**1**^ (10 μg each) for 20 min. After incubation the cells were washed in PBS and new media (2 mL) was added. One coverslip was treated with each preparation and fixed in 4% paraformaldehyde (PFA) in PBS (300 μL), while a further two samples for each preparation were fixed after 20 and 40 min to allow BMDCs time to process the MIS416 conjugates. Coverslips were mounted using ProLong ® Gold with DAPI (Invitrogen). Images were taken using a Zeiss LSM 710 confocal microscope using 340nm, 488nm and 630nm lasers for DAPI, FAM and APC, respectively. Brightfield images were used to identify the shapes of the cells.

A quantification procedure was used to determine the percentage of DCs releasing FAM-SIINFEKL in the cytoplasm, and was carried out by analysing the photomicrographic images. In each image (the total number of images for each condition: A = 4, B = 4, C = 6, D = 4, E = 6, F = 6) the cells were scored as being either DCs releasing FAM-SIINFEKL (green and/or red fluorescent signal in the cell), or DCs that were not releasing FAM-SIINFEKL (yellow fluorescent signal in the cell). The percentage of DCs releasing FAM-SIINFEKL for each condition was then calculated as follows: number of releasing cells/(number of releasing cells + number of non releasing cells) x 100. The results were analyzed with a One Way Anova test corrected for multiple comparisons (Bonferroni) using GraphPad Prism version 6.

### Flow cytometric analysis of DC activation by MIS416 conjugates

1x10^6^ BMDCs were plated in a 24 well plate with 1 mL of complete media in each well, and pulsed with PBS (50 μL) of conjugates **A** or **B** (5 μg), MIS416 (5 μg), SIINFEKL (0.2 ng, Resolving Images), a combination of MIS416 and SIINFEKL, biotin-ttds-SIINFEKL-FAM (0.5 ng) or biotin-SS-SIINFEKL-FAM (0.5 ng). Differing gram amounts of the conjugates were used compared to SIINFEKL so as to give equivalent final molar amounts of SIINFEKL to DCs. Cells were harvested using cold FACS buffer (PBS, 1% BSA, 0.1% NaN_3_) after 4, 12, 24 or 48 h and stained with an infrared near IR-live/dead assay (Invitrogen, 0.05 μL plus 100 μl of FACS buffer for 15 min at 4°C). After washing in FACS buffer, cells were stained with CD11c-APC (clone N418, Biolegend) and H-2Kb-PE/Cy7 bound to SIINFEKL antibody (clone 25D1.16, Biolegend) (1/100 dilution of each antibody plus 100 μL of FACS buffer for 30 min at 4°C) to identify DCs that had SIINFEKL presented on MHC class I. Samples were analyzed using a Gallios flow cytometer (Beckman Coulter, Inc.). In silico analysis was performed using FlowJo software (version 10, TreeStar, Inc.). Cells were gated for singlets (FSC-H vs FSC-A) and DC (SSC-A vs FSC-A). The DC gate was further analyzed for IR-live/dead negative cells, and the expression of CD11c, taking only the healthy live DC population. Results from this analysis showed the percentage of live DCs presenting SIINFEKL on MHC class I. All experiments were repeated at least three times.

BMDCs at day 6 were plated in 6 well plates (1x10^6^, 2 mL of complete media in each well) and pulsed with MIS416 (1, 5, 10 μg), LPS (1 μg), conjugation **A** and **B** (1 μg), (sulfo-nhs-biotin (1 μg) or streptavidin (1 μg) for 24 or 48 h. After incubation, cells were harvested using cold PBS and stained with infrared near IR-live/dead (Invitrogen, 0.05 μL plus 100 μl of PBS for 15 min at 4°C). Cells were then divided into 4 FACS tubes (0.2x10^5^ cells/tube) and stained separately with CD11C-APC antibody (clone N418, Biolegend) and CD40 (clone 3.23, Biolegend) or CD80 (clone 1610, Biolegend) or MHC class II (clone N1MR-4, Cell Lab) (1 μg/10^6^ cells of each antibody in 100 μL of FACS buffer for 15min at 4°C). All marker antibodies were tagged with PE. *FACS analysis*: Samples were analysed using a Gallios flowcytometer (Beckman Coulter, Inc.). Cells were gated for live/dead and DCs (CD11c^+^). The DCs gate was further analysed for expression of activation markers. Results show mean fluorescence intensity (MFI) or the percentage of cells that are positive for the specific activation marker in the case of CD40.

### Preparation of OT-1 T cells

Spleens from OT-1 transgenic mice were collected and cells were separated and filtered through a 100μm filter into a falcon tube (50mL) containing 10mL of PBS. RBC were lysed with 5mL of ammonium chloride buffer (4.15 g NH_4_Cl, 0.5 g KHCO_3_, 0.0186 g EDTA, 500 mL milli-Q water, pH 7.4) for 3 min at 37°C. Cells were washed (300 x g, 5 min), in PBS (20mL) and counted before adding MACS buffer (1X DPBS, 0.5% BSA, 2 mM EDTA, Filter sterilized) and CD8 magnetic beads (Miltenyi Biotec Inc.) (90μL of buffer plus 10μL of beads per 10^7^ cells). Cells were incubated on ice for 30min and washed (300 x g, 5 min) with 1.5mL of MACS buffer per 10^7^ cells. The cells were resuspended in 500μL of MACS buffer per 10^8^ cells and T cells selected on an AutoMACS Pro Separator (Miltenyi Biotec) using the positive selection program according to the manufacturer's instructions. The positive fraction was collected and cells were washed twice in PBS (20mL) (300 x g, 5 min). The percentage of positive CD8+ OT-1 T cells after separation was >95% (data not shown).

### *In vitro* OT-1 T cell proliferation assay

BMDCs at day 5 were plated (5x10^5^ cells/well) in 12 well plates (l mL of complete media each well) and incubated with SIINFEKL (0.5 μg), MIS416 alone (0.5 μg), MIS416 plus SIINFEKL, conjugation **A** (0.5 μg), conjugation **B** (0.5 μg), or biotin-SIINFEKL-FAM (1μg). After 24h of incubation cells were collected, washed in PBS (300 x g, 5 min) and plated (5x10^4^) in 24 well plates (0.5mL of complete media each well) and OT-1 T cells (5x10^5^ in 0.5mL of media) were added. OT-1 T cells were prepared as described previously, and were pre-stained with VPD450 proliferative dye (BD Bioscience). Briefly OT-1 T cells were resuspended (1x10^6^/mL) in PBS and VPD450 was added to a final concentration of 1mM. Cells were then incubated at 37°C for 10 min and washed 3 times in PBS (300 x g, 5 min) before adding them to DC cultures. After 48 or 72 h of incubation cells were harvested and stained with infrared near IR-live/dead (Invitrogen, 0.05 μL plus 100 μl of PBS for 15 min at 4°C). After washing in PBS, cells were stained in FACS buffer with CD8 (clone 53–6.7, Cell Lab- Beckman Coulter, Inc.) and CD69 (clone H1.2F3, Cell Lab) (1μg/10^6^ cells of each antibody in 100 μL of FACS buffer for 15min at 4°C). Samples were analysed using a Gallios flowcytometer (Beckman Coulter, Inc.). In silico analysis was performed using FlowJo software (version 9, TreeStar, Inc.). The cells were gated for singlets (FSC-H vs FSC-A), live/dead and CD8^+^. The CD8^+^ gate was further analysed using the proliferation software tool in FlowJo version 9 in order to calculate the percentage of proliferating CD8+ OT-1 T cells in each sample.

## Supporting Information

S1 File Figure A. Schematic of the conjugation strategy used to couple biotinylated molecules to MIS416. **Figure B. Schematic representation of conjugate A^1^. Figure C. Calibration curve of streptavidin-PE** (excitation 500nm, emission 570nm). **Figure D. Demonstration of linkage of NHS-biotin to MIS416.** The graph shows the percentage of streptavidin attached to MIS416, calculated by dividing the total amount of fluorophore coupled to MIS416 over the total amount of fluorophore in the solution before the reaction. Error bars represent SEM. The experiment was repeated four times. **Figure E. Calibration curve of biotin-SS-SIINFEKL-FAM and biotin-ttds-SIINFEKL-FAM** (excitation 488nm, emission 520nm). Figure F. Evaluation of activation marker expression on DCs after treatment with conjugation A and B. **Table A. Amount of SIINFEKL conjugated to MIS416 in conjugates A and B. Table B. Conjugates used in this study.**
(DOCX)

## Abbreviations

APC: antigen presenting cell
DC: dendritic cell
OVA: ovalbumin
SAV: streptavidin
MHC: major histocompatibility complex
NOD-2: nucleotide-binding oligomerization domain containing 2
TLR-9: toll-like receptor-9
PEG: polyethylene glycol
FITC: fluorescein isothiocyanate
TCEP: tris(2-carboxyethyl)phosphine
SEM: standard error of the mean
FAM: fluoresceinamine
PBS: phosphate buffered saline
