# Supplementary material for: Bridging Small Molecules to Modified Bacterial Microparticles Using a Disulphide Linkage: MIS416 as a Cargo Delivery System
Source: PLoS One. 2015 Dec 22;10(12):e0145403. doi: 10.1371/journal.pone.0145403 (PMC4687933; doi:10.1371/journal.pone.0145403)
Supplement: S1 File — Figure B. Schematic representation of conjugate A1. Figure C. Calibration curve of streptavidin-PE (excitation 500nm, emission 570nm). Figure D. Demonstration of linkage of NHS-biotin to MIS416. The graph shows the percentage of streptavidin attached to MIS416, calculated by dividing the total amount of fluorophore coupled to MIS416 over the total amount of fluorophore in the solution before the reaction. Error bars represent SEM. The experiment was repeated four times. Figure E. Calibration curve of biotin-SS-SIINFEKL-FAM and biotin-ttds-SIINFEKL-FAM (excitation 488nm, emission 520nm). Figure F. Evaluation of activation marker expression on DCs after treatment with conjugation A and B. Table A. Amount of SIINFEKL conjugated to MIS416 in conjugates A and B. Table B. Conjugates used in this study. (DOCX) [file pone.0145403.s001.docx]

**Supplementary data**


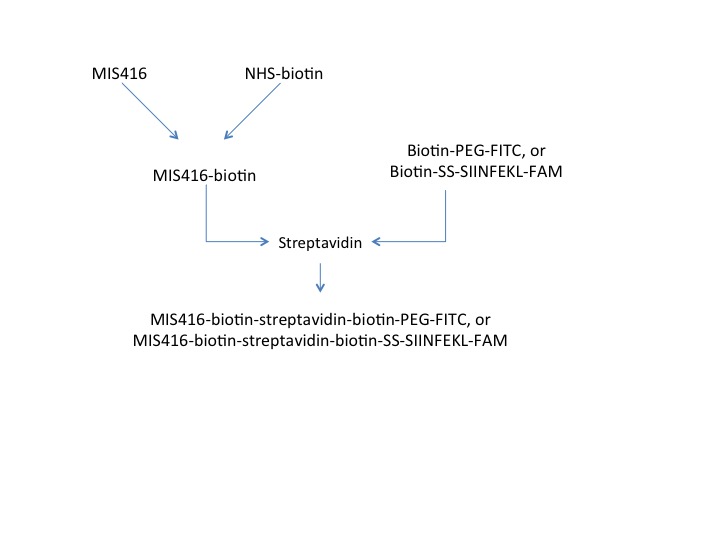


Figure A. Schematic of the conjugation strategy used to couple biotinylated molecules to MIS416.

Streptavidin was used to bridge MIS416-biotin to a biotinylated small molecule. Biotinylation of MIS416 was carried out in a reaction with NHS-biotin. Either Biotin-PEG-FITC, or Biotin-SS-SIINFEKL-fluoresceinamine (FAM) were used as small molecules to attach to MIS416-biotin in this study. PEG, polyethylene glycol; FITC, fluorescein isothiocyanate; SIINFEKL peptide; FAM, fluoresceinamine.


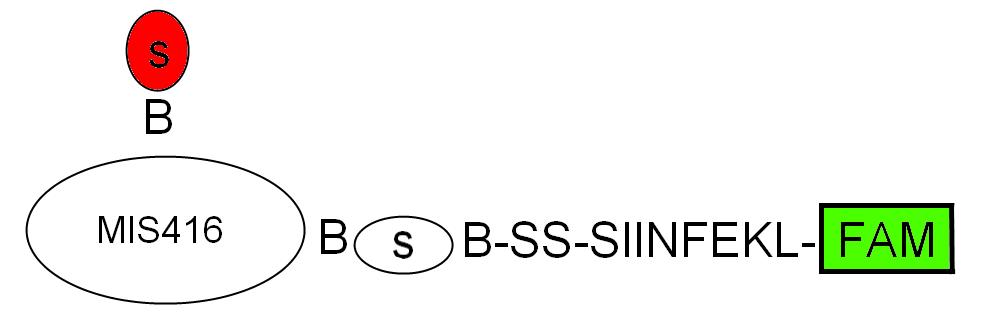


**Figure B. Schematic representation of conjugate A^1^**.

Conjugates A and B were modified by the addition of streptavidin-allophycocyanin (SAV-APC) to make conjugates A^1^ and B^1^. The figure shows a schematic representation of conjugate A^1^. MIS416 microparticles in conjugates A^1^ and B^1^ were fluorescently labelled with allophycocyanin (S, indicated in the red oval), while the attached cargo, SIINFEKL-FAM, was labelled with fluoresceinamine, (FAM, indicated in the green box).


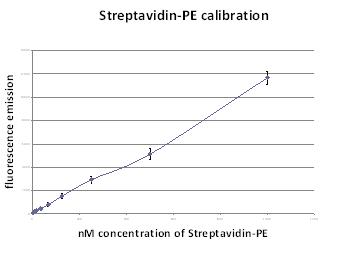


**Figure C**. **Calibration curve of streptavidin-PE** (excitation 500nm, emission 570nm).

R^2^=0.745


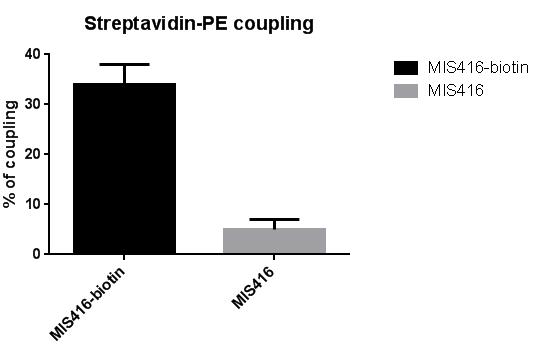


**Figure D.** **Demonstration of coupling of NHS-biotin to MIS416**. This graph represents the percentage of streptavidin coupled in 4 separate experiments. The % of coupling has been calculated dividing the total amount of fluorophore coupled to MIS416 to the total amount of fluorophore in the solution before the reaction. Error bars represent SEM.

**Figure E.** **Calibration curve of biotin-SS-SIINFEKL-FAM and biotin-ttds-SIINFEKL-FAM** (excitation 488nm, emission 520nm).

R^2^ value for biotin-SIINFEKL-FAM=0.92

R^2^ for biotin-SS-SIINFEKL-FAM=0.99


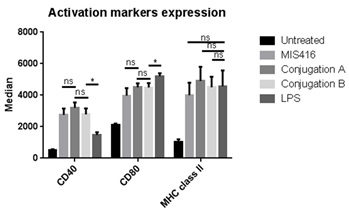


Figure F. Evaluation of activation marker expression on DCs after treatment with conjugation A and B

DCs (1x10^6^ in 2 mL of media) were treated with MIS416, LPS (1 μg), and conjugation **A** and **B** (1 μg each). Cells were collected and stained in live/dead assays and with different antibodies to detect activation markers (CD40, CD80, MHC class II) and DCs (CD11c). The concentration used for antibodies was 1 μg/10^6^ cells of each antibody in 100 μL of FACS buffer for 15min at 4°C. Y axis represents the Median. This experiment was repeated three times

**Table A: Amount of SIINFEKL conjugated to MIS416 in conjugate A and B**

| Conjugate | Mass of MIS416 | Total mass of SIINFEKL-FAM coupled to MIS416 | Amount of SIINFEKL-FAM coupled to 10 μg of MIS416 (nmol) |
| --- | --- | --- | --- |
| Conjugate **A**  MIS416-biotin-streptavidin-biotin-SS-SIINFEKL-FAM | 200 μg | 6.3 μg | 0.171 nmol |
| Conjugate **B**  MIS416-biotin-streptavidin- biotin-ttds-SIINFEKL-FAM | 200 μg | 5.7 μg | 0.158 nmol |

**Table B: Conjugates used in this study**

| Conjugate | Long name | Used in Figure: |
| --- | --- | --- |
| Conjugate **A** | MIS416-biotin-streptavidin-biotin-SS-SIINFEKL-FAM | 3,5,6 |
| Conjugate **B** | MIS416-biotin-streptavidin- biotin-ttds-SIINFEKL-FAM | 3,5,6 |
| MIS416/FITC | MIS416-biotin-streptavidin- biotin-PEG-FITC | 2 |
| Conjugate **A^1^** | APC-SAV-biotin-MIS416-biotin-streptavidin-biotin-SS-SIINFEKL-FAM | 4 |
| Conjugate **B^1^** | APC-SAV-biotin-MIS416-biotin-streptavidin- biotin-ttds-SIINFEKL-FAM | 4 |
